# Indents

**Published:** 1910-12-15

**Authors:** 


### INDENTS.

☞Brief Articles of Technical or Scientific Value will receive notice and comment. Contributions invited.

*Sodoxylin.* In the treatment of pyorrhea or gingivitis too often failures are due to the fact that the dentist has been working only on the surface. Talbot and others believe these troubles have a deep-seated origin—are due to a constitutional defect—which must be dealt with before results can be expected. It may be that these diseases originate as they think in the very bowel itself, where, under certain conditions, food waste accumulates, ferments, and gives rise to complex bacterial poisons. These poisons are in the nature of fatty acids; they are absorbed into the blood-stream and, in the course of time, surcharge every part of it. Not only the blood but the secretions also become loaded with them. The mucus and saliva of the mouth become highly acid (even corrosive) and lose their natural protective powers.

Talbot suggests as a remedy Sodoxylin—a mixture of sodium sulphocarbolate, sodium sulphate, sodium bicarbonate, colchicine, juglandin, xanthoxylin and aromatics. This helps to eliminate the waste matter in which the bacterial poisons breed; neutralizes the acidity and checks the formation of pathogenic bacteria.

Sodoxylin internally and Sodoglycerote applied to the gums twice a week is a successful treatment for gingivitis and pyorrhea.The patients co-operation is necessary; he should be instructed to use a mouth wash two or three times daily, with gum massage by means of the tooth-brush.

*Calmine.* This is a new preparation sold under this trade name. Chemically it is said to be sodium diethylbarbiturate. It is a fine white powder soluble in water, having an alkaline taste, It is made in

England, but marketed in this country by the Abbott Alkoloidal Co., in powder form and in two and one-half grain tablets. It is claimed to be a succedaneum for the older hypnotics and analgesics, but with none of their usual disagreeable secondary effects. It is said to be very useful in insomnia, nervous irritability, hysteria, mania, wakefulness, mental overstrain and all forms of nervous irritability. It ought to be a good preparatory treatment for painful or exhausting operations, and for securing rest periods in prolonged neuralgias and odontalgia.

*The Difference Between Morphine and Codeine and Heroin.* A short time ago the Board of Health of the city of New York, promulgated an ordinance providing that "No cocaine or salt of cocaine, and no morphine or salt of morphine, either alone or in combination with other substances, shall be sold at retail by any person in the city of New York, except upon the prescription of a physician." Immediately every druggist in the city stopped the sale of all preparations containing any derivative of opium and raised such a furore, that the Acting Commissioner of the Board of Health felt called upon to explain what every druggist ought to have known, viz: that "Heroin and Codeine are not salts of morphine, and therefore are not included in the proscribed list."

In order to make this mattter perfectly clear, the following on the subject of opium is submitted for the information of the many who have been laboring under the misapprehension that Codeine and Heroin are salts of opium or of morphine.

Opium, besides wax, fat, glucose, gum, pectin, resin, etc., contains about 20 alkaloids, among them being Morphine, Codeine, Thebaine, Narceine, Papaverine, Pseudomorphine, Narcotine, etc., all occurring in varying amounts according to the grade of opium. While Morphine is an analgesic, it does not follow that Trebaine is an analgesic simply because it is also derived from opium. One might equally as well say that Acetanilid and Diamond Dyes have similar therapeutic effects, because both are derived from coal tar. Heroin, as is well known to every druggist, is a synthetic preparation and is not an alkaloid of opium. There are no salts of opium; there are active principles or alkaloids from which by the addition of acids, salts are formed which become, not salts of opium, but salts of morphine, salts of codeine, etc. All chemists know this and all druggists probably know it, but fear of transgressing the law made the New York druggists take a position contrary to that which their knowledge of chemistry would indicate to be the correct one. Codeine and Heroin are not salts, either of opium or of morphine, the one being an active principle, and the other a synthetic compound. Furthermore, Morphine and Codeine have widely different properties; Codeine being entirely devoid of the evil effects of Morphine, not locking up the secretions or causing constipation; and the Codeine habit is a thing unknown in medical literature. In fact, all authorities agree that Codeine does not create habit.

From all the above we glean the following facts:

1. Opium and derivatives of Opium, except Morphine and its salts are not in the proscribed list under the Regulation of the New York Board of Health.

2. Codeine and Heroin are not salts of Opium.

3. Codeine and Heroin are not salts of Morphine.—*Apothecary and New England Druggist, Oct. 1910.*

*Duralumin, a New Aluminium Alloy.* Duralumin is a new alloy of aluminium discovered by H. B. Weeks, head chemist at Vickers' Sons and Maxims' Works, Barrow, England, This alloy is a little heavier than pure aluminium, but is as strong as steel. Weeks declares the alloy can be rolled, drawn, stamped, and extended or forged at suitable temperatures. It is much less easily corroded than other aluminium alloys, and has only one-third the weight of brass.—*Public Ledger*, Philadelphia.

*Electric Dental Engine Care.* It should not be forgotten that the bearings of a dental engine needs lubrication. The foot-power engine speaks for itself by demanding more muscular effort, but the electric may suffer severely and give no sign. It is, however, a delicate machine and should not be neglected nor left to the tender mercies of the office boy or girl. A little, and as frequent as necessary should be the rule in oiling, and about once in three months a drop or two of absolute alcohol or benzine put into the oilcups to thoroughly clean them out is good practice. Be careful to keep oil from the commutator brushes, they should be kept quite clean, especially if the motor is a low voltage machine.—Trueman in *Dental Brief.*

*Labeling Bottles, Etc.* A very handy label which is always ready for instant use may be found in the rolls of ready-gummed paper which comes in half-inch and inch widths, and are of considerable length. They may be obtained at art stores, and are classed with passe-partout material. A strong, smooth white variety should be selected. Cut from the roll a length sufficient to pass around the bottle and lap over about half an inch. Moisten the paper, attach one end to the bottle, hold it firmly and wind the paper round the bottle and lap it. Such a label will last and stick fast when a label gummed to one side of the bottle only, will become loosened and slip off at the first touch. Another handy material for labels is rubber adhesive plaster, to be had in any drug store, it comes in small ten-cent packages, one package is sufficient for a dozen or more labels of ordinary size. If the bottle is slightly warmed before applying the label, and the label rubbed well into contact, the bottle may be washed or even soaked on the outside without fear of the labels coming off. The lettering should be done with water proof ink, Higgin's Waterproof India Ink, as prepared for architect's use, is satisfactory. Trueman in *Dental Brief.*

*The Public Conscience Awakening on Preventable Disease.* The public interest in health conditions is rapidly increasing. Evidence of this fact can be found in the changing attitude of newspapers toward public health problems and the growing comprehension of their importance. For instance, the recent editorial comment in the *New York World* on typhoid fever would have been impossible ten years ago.

"Every life lost by typhoid is a wasted life. It is absolutely preventable. People who live in marble halls without caring whether poison runs in the pipes behind them; the very rich who spend millions in display, but neglect sanitation; college professors caught unaware by epidemics like that in Ithaca—these have themselves to blame if the disease occurs. Typhoid originating in any community disgraces it."

In former generations, epidemics of typhoid fever and other filth diseases have been regarded as dispensations of Providence just as a high death rate has been looked on as a natural ratio which could not be altered. The *World* has emphasized one of the most important principles of the coming gospel of health—*i. e.*, that the occurrence of preventable diseases is a discredit to the community.—*A. M. A. Journal.*

*Two Remedies For Race Suicide.* "The best crop is the baby crop," said Mr. Roosevelt at Ames, Iowa, recently. True, but what shall it profit a nation if it produce ever so large a crop of babies but fail to keep them alive until they grow to years of maturity and usefulness? That nation is the strongest which has the largest number of mature, healthy and capable men and women. Mr. Roosevelt has done great service in decrying race suicide and urging a larger birth rate. There are, however, two ways in which a nation can annihilate itself. One is by a diminishing birth-rate and the other is by an increasing death-rate among infants and young adults. The child which dies before it reaches maturity is a dead loss to the community. If Mr. Roosevelt will take up with his accustomed vigor, the conservation of existing lives, in conjunction with his campaign for an increase in the number of new lives, as a combined remedy for race suicide, he will aid a growing and inevitable reform and will vastly increase the obligations of the country to him for his services.—*Journal A. M. A.* September 17, 1910.

*Prophylaxis for Infants.* The practical application of our accumulated scientific knowledge with regard to the human mouth as a focus of infection in relation to the prophylaxis of those diseases which distinctly have their source of origin in mouth infection—and among these must be included tuberculosis and pneumonia—can never be successfully accomplished until all of the forces and agencies now at work in the endeavor to solve the problem of higher vital standards in the nation have come to realize the necessity for oral prophylaxis, and have adopted means for educating the public up to its practical realization.

One other aspect of this broad question which is brought out by the Census Bureau report relates particularly to the question of the high rate of infant mortality. It is a notable fact that the relatively high death-rate among children occurs distinctly during the first dentitional period. It has also been definitely proved, in so far as clinical evidence and logical reasoning from clinical evidence can arrive at a definite conclusion, that difficult or disordered dentition may and does so disarrange the nervous organizations of the infant and young child as to seriously disturb its nutritional mechanism, reduce its vital resistance, and make it an easy subject for the invasion of disease-producing organisms which set up their pathogenic activities in the gastro-intestinal tract and produce that class of disorders which are characteristic of the dentitional period, and which are responsible for the high rate of mortality everywhere recorded in connection with the early years of existence.

We are firmly of the opinion that the high rate of child mortality will never be materially reduced until those who are charged with the responsibility of mitigating the conditions which surround the growing infant are made to clearly comprehend the relationship between difficult dentition and infantile disorders, and to learn by practical experience the beneficient and life-saving virtues of the gum lancet when intelligently and skillfully used in these cases.

It is in the foregoing aspects of the question that the members of the dental profession everywhere are concerned. It is they who are in position by their special training and by their personal influence to bring these matters to the attention of physicians and of the authorities now fully awake to the necessity for active work in reducing the mortality figures of our country. The end sought cannot be accomplished by talk or arguments based upon glittering generalities, but by the individual effort of those who have an intelligent comprehension of the etiology pathology and prophylaxis of the disorders here under consideration. Dr. E. C. Kirk, in October *Cosmos.*

